# Platelet-Rich Plasma and Related Orthobiologics for the Treatment of Equine Musculoskeletal Disorders—A Bibliometric Analysis from 2000 to 2024

**DOI:** 10.3390/vetsci11080385

**Published:** 2024-08-21

**Authors:** Jorge U. Carmona, Luis H. Carmona-Ramírez, Catalina López

**Affiliations:** 1 Grupo de Investigación Terapia Regenerativa, Departamento de Salud Animal, Universidad de Caldas, Manizales 170004, Colombia; 2 Grupo de Investigación EFE, Facultad de Educación, Universidad Católica de Manizales, Manizales 170004, Colombia; lucarmona@ucm.edu.co; 3 Grupo de Investigación Patología Clínica Veterinaria, Departamento de Salud Animal, Universidad de Caldas, Manizales 170004, Colombia; catalina.lopez@ucaldas.edu.co

**Keywords:** horse, platelet-rich plasma, platelet lysate, autologous protein solution, autologous conditioned serum, IRAP, osteoarthritis, tendonitis, desmitis, laminitis

## Abstract

**Simple Summary:**

Platelet-rich plasma and related orthobiologics, such as platelet lysates, autologous conditioned serums, and autologous protein solutions, are relatively new regenerative therapies used in the medical management of chronic musculoskeletal disorders in horses. Numerous papers have been published in this area; however, there is no information on the bibliometric impact of these papers in the published veterinary literature. A bibliometric analysis was performed using the bibliometrix R package to evaluate the documents registered in the WOS and Scopus databases from 2000 to 2024. The obtained registers were evaluated considering their overview, sources, authors, documents, words, trending topics, clustering, conceptual structure, and social structure. *Frontiers in Veterinary Science*, *Journal of Equine Veterinary Science*, *BMC Veterinary Research*, and the *American Journal of Veterinary Research* were the most used sources to publish these papers. Universidad de Caldas, Colorado State University, University of California-Davis, and University of Leipzig were the most productive institutions, while the USA, Brazil, and Colombia had the highest production in this field. The most frequently used keywords were horse, platelet-rich plasma, equine, osteoarthritis, and autologous conditioned serum. The trending topics in this field are platelet lysates and orthobiologics. The collaborative network of authors, institutions, and countries is clustered and isolated in small research groups.

**Abstract:**

(1) Background: There is increasing interest in the use of platelet-rich plasma and related orthobiologics for the treatment of chronic musculoskeletal disorders in horses; however, there is no information on the bibliometric impact of the literature published in this area. (2) Methods: A bibliometric analysis was performed using the bibliometrix R package by analyzing the documents registered in the WOS and Scopus databases from 2000 to 2024. The included registers were evaluated according to the menu of results from the biblioshiny web app (overview, sources, authors, documents, words, trending topics, clustering, conceptual structure, and social structure). (3) Conclusions: The documents produced were mainly published in *Frontiers in Veterinary Science*, *Journal of Equine Veterinary Science*, *BMC Veterinary Research*, and the *American Journal of Veterinary Research*). The most productive institutions were Universidad de Caldas, Colorado State University, University of California-Davis, and University of Leipzig, and the most productive countries were the USA, Brazil, and Colombia. Horse, platelet-rich plasma, equine, osteoarthritis, and autologous conditioned serum were the most frequently used keywords. The trending topics in this area are platelet lysates and orthobiologics. The collaboration network of authors, institutions, and countries shows an isolated development of individual author networks with modest collaboration between institutions and countries.

## 1. Introduction

Regenerative medicine is a growing field of medical science (in both human and veterinary medicine) that seeks to explore different therapeutic approaches based on the clinical use of cells, proteins (namely growth factors (GFs) and cytokines), and even gene editing to regenerate cells, tissues, or organs affected by congenital, traumatic, ischemic, and chronic inflammatory diseases, among others, in pediatric to geriatric patients [1,2,3,4,5].

Chronic musculoskeletal diseases, such as osteoarthritis (OA), and inflammatory/degenerative conditions of tendons and ligaments are common in humans [6,7,8] and horses [1,2,9,10], causing pain (clinically manifested by lameness in horses) and functional loss of mobility, affecting the quality of life of patients and limiting their productivity [11,12].

Over the past 24 years, several therapeutic approaches based on the concept of “regenerative medicine” have become real options in the field of equine orthopedics [1,5,13,14,15]. These regenerative therapeutic strategies generally include the use of pure bone marrow aspirates [16,17,18], stem cells [19,20,21,22], and orthobiologics such as platelet-rich plasma (PRP) [23,24], platelet lysates [25,26], autologous protein solution (APS), and autologous conditioned serum (ACS) [27,28,29,30,31,32,33]. It should be noted that horses are one of the most important animal models for studying the pathophysiological mechanisms of chronic musculoskeletal disorders and for evaluating the response to experimental treatments that could potentially be used in humans. Therefore, the results observed in horses treated with this type of orthobiologics may apply to humans [34,35].

Currently, the use of hemocomponent-based orthobiologics for the treatment of chronic musculoskeletal disorders in horses may be more popular than the use of stem cells because they are easier to obtain and their cost is low compared to stem cell treatments [36]. Although there are no specific guidelines for the use of regenerative medicine-based medications in horses [1], these types of orthobiologics are generally used as first-line treatments, with the use of stem cells reserved for refractory cases [36,37].

According to an extensive literature search, there are no published studies evaluating the bibliometric impact on the use of PRP and related orthobiologics for the treatment of chronic musculoskeletal disorders in horses. Therefore, the aims of the present study were (1) to know the chronological bibliometric development of peer-reviewed publications developed in the field of PRP and related orthobiologics for the treatment of chronic musculoskeletal diseases in horses from 2000 to the present; (2) to determine how this bibliometric body is shaped and influenced by key authors, institutions, and countries; and (3) to identify the use of most common keywords, trending topics, clustering, conceptual structures, and social structures related to the topic.

The relevance of this bibliometric review stems from the fact that there are currently no published studies evaluating the quantity and quality of the papers published in this research field. Therefore, the use of bibliometrics allows us to evaluate how academic works influence and are related to the development of a specific research field in terms of credibility, quality, and impact. In addition, this type of review is useful to evaluate the state of the art, hotspots, trends, and frontiers of specific research topics [38].

## 2. Materials and Methods

### 2.1. Database Selection and Keyword Search

Web of Science (WOS) (Clarivate Analytics, London, UK) and Scopus (Elsevier B.V. Amsterdam, The Netherlands) were selected for this scientometric study. WOS allows for searching more than 12,000 journals published in 45 languages in various fields of science. Scopus includes more than 27,950 active series. Both databases allow bibliometric analysis, including citation tracking, citation counts, and H-index calculations, among other citation analysis tools [39,40].

### 2.2. Search Equation and Filter Application

The following search equation was used for this bibliometric analysis:

(“platelet-rich plasma” or “platelet concentrate” or “platelet lysate” or “platelet supernatant” or “platelet lyophi*” or “autologous protein solution” or “autologous conditioned serum”) and (horse* or equine or equus or equid*) and (osteoarthritis or “degenerative joint disease*” or “joint disease*”) or “joint disease*” or joint* or arthritis or cartilage* or chondro* or “joint degeneration” or tendinitis or tendinopathy or desmitis or desmopathy or laminitis).

After obtaining the results of each database, they were first filtered by subject area (Veterinary Sciences only (WOS) or Veterinary Sciences (Scopus)) and by year (2000–2024).

### 2.3. Register Screening and Selection Criteria

The registers retrieved from each database were merged using the R library *(library(bibliometrix))*, by using the *merge()* function, which was programmed to detect duplicate documents. After receiving this preliminary database, J.U.C. and C.L. reviewed them individually for further screening, taking into account the title, keywords, and abstract of each registry and evaluating whether the selected registries were effectively related to the search area evaluated in the bibliometric analysis. The final decision to exclude registries was made by consensus among the researchers. The types of documents considered for this bibliometric analysis were articles and reviews, according to the biblioshiny web application [41]. Documents such as proceedings papers, book chapters, conference papers, editorial materials, notes, preprints, and early access were not included.

### 2.4. Data Analysis

The data from the definitive database were analyzed with the R package bibliometrix (R4.4.0) [41] and visualized with the biblioshiny web app to perform bibliometric processing and visual knowledge map analysis, taking into account the general menu of the app, which includes overview, sources, authors (authors, affiliations, and countries), documents, words, trending topics, clustering, conceptual structure, and social structure.

## 3. Results

From a total of 561 documents, 258 registers were obtained from WOS, and 303 documents were obtained from SCOPUS. Subsequently, the *merge()* function of the R package allowed for the exclusion of 152 duplicate documents, resulting in a total of 409 records. Then, 180 registers were discharged after manual revision, for a total of 229 validated documents.

The records discharged were those that did not reach the requirements to be included in this study, since their title, abstract, and keywords were not related to the research field evaluated.

**Figure 1:**
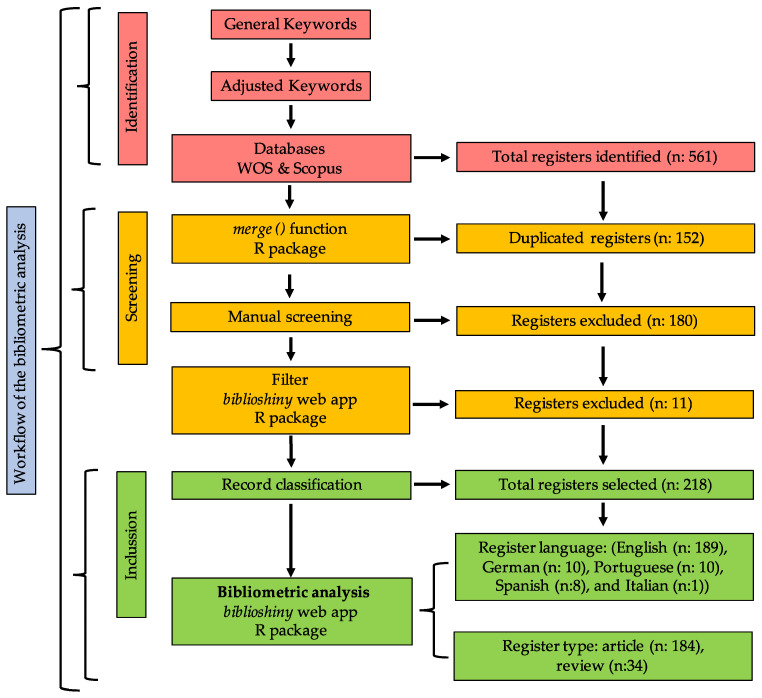
Workflow of the study and results of the registers selected for the bibliometric analysis.

This revised database was uploaded to the biblioshiny web app to refine the registers by excluding documents that were not considered articles or reviews. Finally, a total of 218 registers were obtained for the bibliometric analysis, of which 189 documents were written in English, 10 in German, 10 in Portuguese, 8 in Spanish, and 1 document was published in Italian. It should be noted that 184 registers corresponded to articles and 34 to reviews (Figure 1).

### 3.1. Overview

The first registered document describing a procedure for obtaining equine platelet-rich plasma using a semi-automated kit was published in 2004 [42]. From that moment until the present, the scientific production in this field has been researched in 59 published sources (journals), with an annual growth rate of 9.37% and an average age per document of 7.39 years. In addition, the average number of citations per document was 16.89, with a total number of references used of 1212.

**Figure 2:**
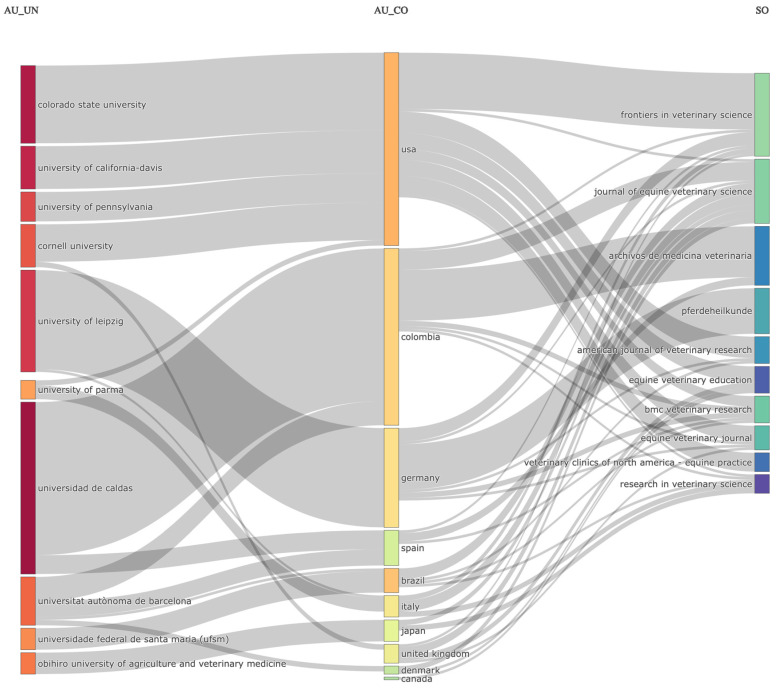
Three-field plot of the connections from the top 10 institutions (left) with the top 10 countries (middle) and the top 10 sources of papers on PRP and related orthobiologics for the treatment of chronic musculoskeletal diseases in horses from 2000 to 2024. AU_UN: author institution; AU_CO: author country; SC: source (journal).

A total of 715 authors have written in this field, with an average of 4.99 authors per document. The percentage of international co-authorships was 2.74%. The authors used a total of 421 keywords. The connections of countries to journals and institutions regarding the scientific production of platelet-rich plasma and related orthobiologics for the treatment of equine chronic musculoskeletal diseases from 2000 to 2024 is shown in Figure 2.

### 3.2. Sources

The top 10 most relevant journals (sources) in which articles or reviews on PRP and related orthobiologics have been published were *Frontiers in Veterinary Science* (26 registers), *Journal of Equine Veterinary Science* (24 registers), *BMC Veterinary Research* (11 registers), *Equine Veterinary Journal* (11 registers), *Pferdeheilkunde* (11 registers), *American Journal of Veterinary Research* (11 registers), *Archivos de Medicina Veterinaria* (10 registers), *Veterinary Clinics of North America* (8 registers), *Equine Veterinary Education* (7 registers), and *Research in Veterinary Science* (7 registers).

Table 1 shows the rank, frequency, cumulative frequency, and zone of the top ten sources according to Bradford’s law.

**Figure 3:**
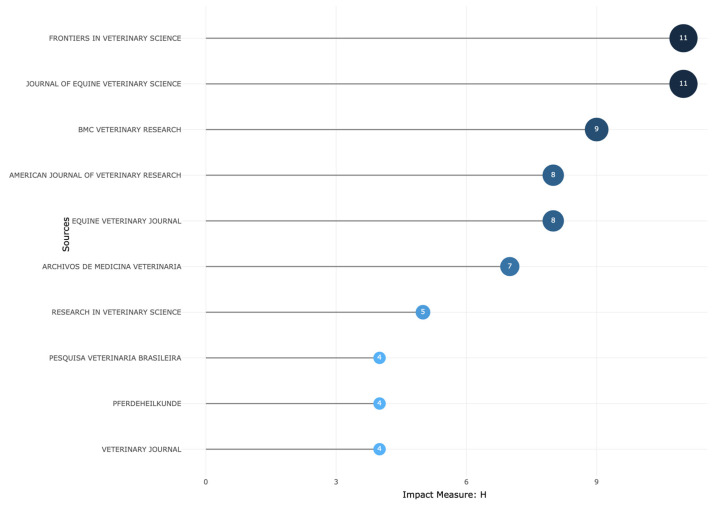
Top 10 journals local impact as measured by H-index that have published articles and reviews on PRP and related orthobiologics as treatments for equine musculoskeletal disorders (2000–2024).

Figure 3 shows the local impact (measured by the H-index) of the top 10 sources (journals) over the observation window (2000–2024) on this topic.

### 3.3. Authors

According this bibliometric analysis, the top 10 most relevant authors on this topic are Carmona, J. (29 registers), López, C. (18 registers), Prades, M. (10 registers), Frisbie, D. (8 registers), Giraldo, C. (8 registers), Ortved, K. (7 registers), Seo, J. (7 registers), Tablin, F. (7 registers), Conti V, (6 registers), and Lischer, C. (6 registers).

**Figure 4:**
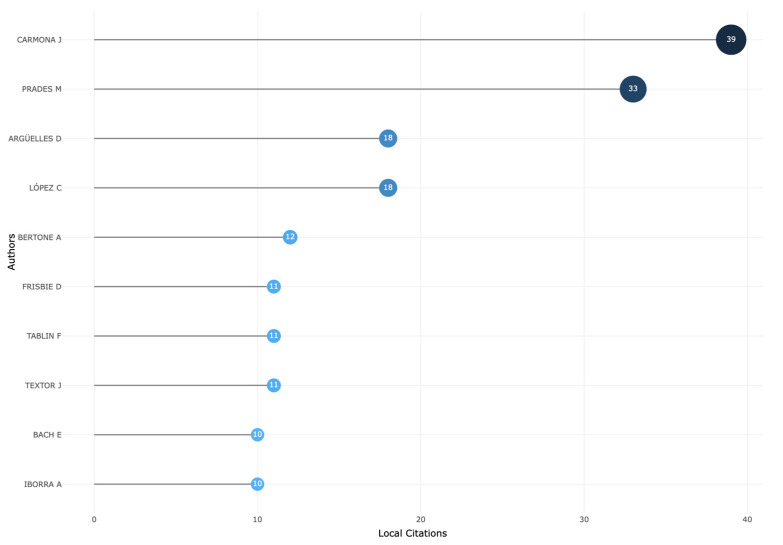
Top 10 most cited local authors who published articles and reviews on PRP and related orthobiologics as treatments for equine musculoskeletal disorders (2000–2024).

**Figure 5:**
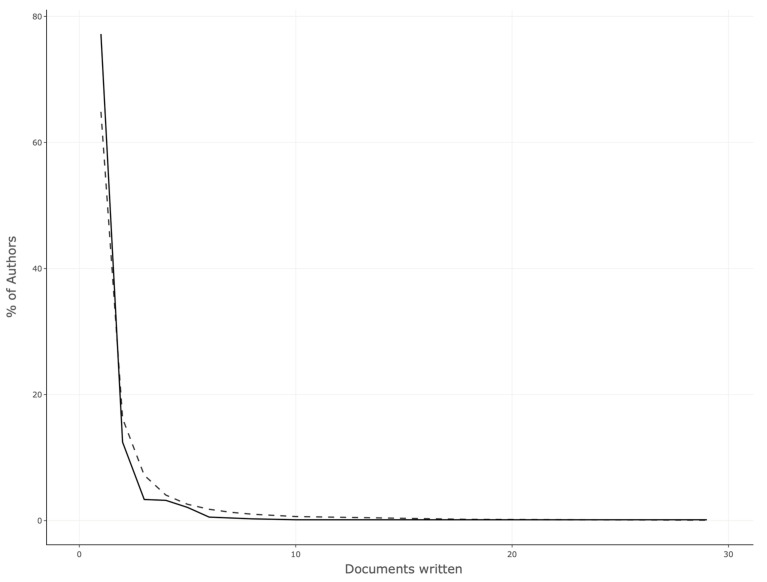
Author productivity measured with Lotka’s law.

**Figure 6:**
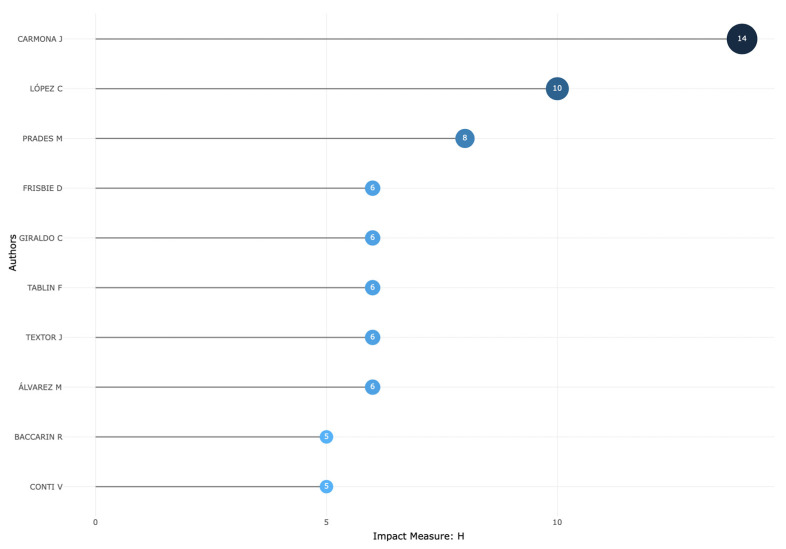
Top 10 authors’ local impact as measured by H-index, who have published articles and reviews on PRP and related orthobiologics as treatments for equine musculoskeletal disorders (2000–2024).

On the other hand, the top 10 most local cited authors are shown in Figure 4. The bibliometric productivity on the evaluated topic of this work according to Lotka’s law is presented in Figure 5, while the author’s impact measured by the H-index is shown in Figure 6.

The top 10 most relevant affiliations were Universidad de Caldas, Colombia (39 registers); Colorado State University, USA (20 registers); University of California-Davis, USA (16 registers); University of Leipzig, Germany (16 registers); Obihiro University of Agriculture and Veterinary Medicine, Japan (11 registers); Cornell University, USA (10 registers); Universitat Autònoma de Barcelona, Spain (10 registers); University of Pennsylvania, USA (10 registers); Universidade Federal de Santa Maria, Brazil (8 registers); and Universidade Federal de Viçosa, Brazil (6 registers).

The top 10 countries that produced the largest quantity of registers about this bibliometric subject study were the USA (83 registers), Brazil (47 registers), Colombia (38 registers), Germany (30 registers), Italy (19 registers), UK (13 registers), Japan (12 registers), Spain (9 registers), Denmark (5 registers), and Canada (4 registers).

### 3.4. Documents

Table 2 shows the most cited documents worldwide according to this bibliometric analysis.

### 3.5. Words

**Figure 7:**
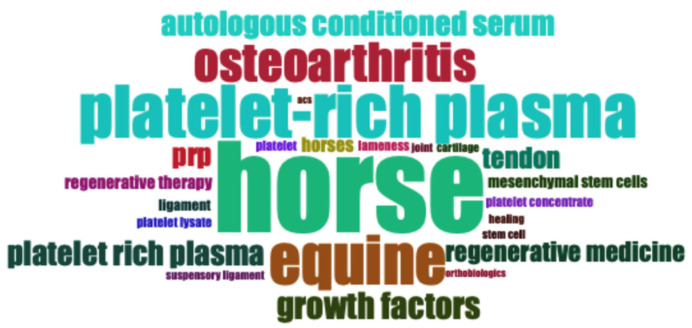
Top 25 most used keywords over time (2000–2024).

The top 10 most frequent keywords used were horse (81 occurrences), platelet-rich plasma (47 occurrences), equine (44 occurrences), osteoarthritis (35 occurrences), growth factors (24 occurrences), autologous conditioned serum (22 occurrences), platelet rich plasma (22 occurrences), prp (20 occurrences), tendon (19 occurrences), and regenerative medicine (18 occurrences). The 25 most common keywords used in the documents included in this bibliometric analysis are shown in Figure 7.

### 3.6. Trending Topics

**Figure 8:**
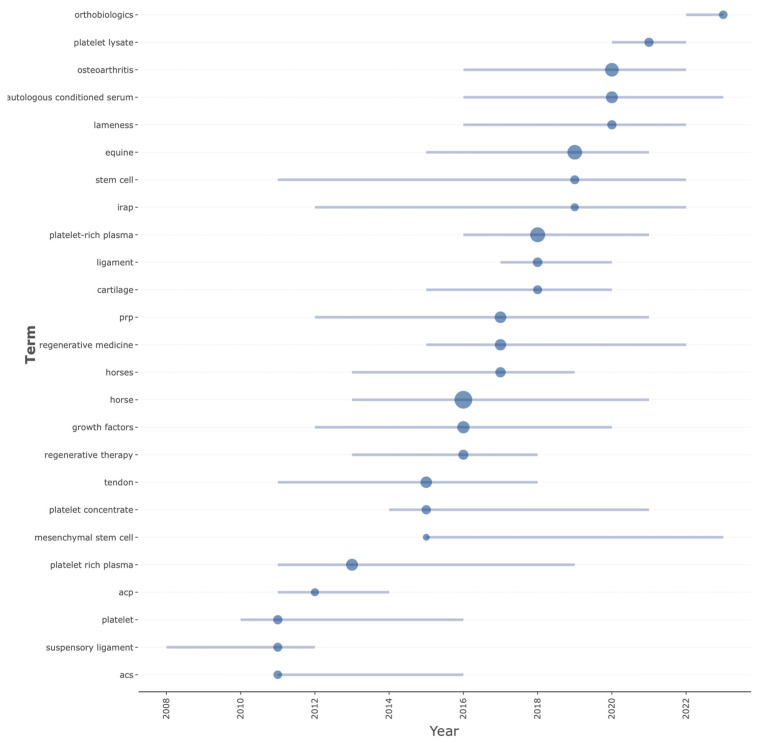
Trending topics in author keywords over time on PRP and related orthobiologicals as treatments for equine musculoskeletal disorders (2000–2024).

The evolution of trending topics according to the author keywords on the field of this bibliometric analysis is shown in Figure 8. The top five trending topics include platelet-rich plasma, autologous conditioned serum, osteoarthritis, platelet lysate, and orthobiologics.

### 3.7. Clustering

The coupling analysis of the documents of the evaluated topics by references, considering the global citation score, and the cluster labeling by author keywords revealed three dominant clusters that provide key insights into the major themes and research trends in this research area. Cluster 1 includes the keywords “gel and growth factors”.

Cluster 2 focuses on “osteoarthritis, platelet-rich plasma, and autologous conditioned serum”. Cluster 3 focuses on “platelet-rich plasma, bone marrow, and mesenchymal stem cells”. This last cluster has the highest centrality (3.31) and the largest impact (1.61).

### 3.8. Conceptual Structure

**Figure 9:**
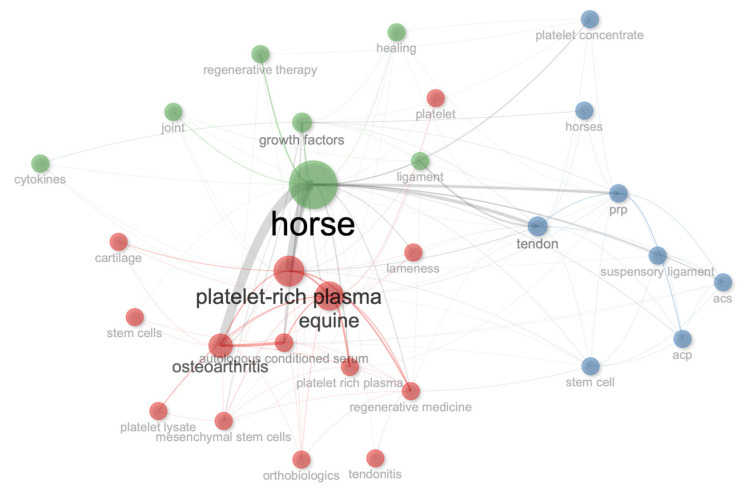
Co-occurrence network analysis of author keywords.

Co-occurrence network analysis of the author keywords revealed four clusters mainly related to the keywords horse (cluster 1, betweenness: 495.85, closeness: 0.020, and page rank: 0. 147), platelet-rich plasma (cluster 2, betweenness: 48.62, closeness: 0.015, and page rank: 0.069), and osteoarthritis (cluster 2, betweenness: 42.20, closeness: 0.014, and page rank: 0.057) (Figure 9).

**Figure 10:**
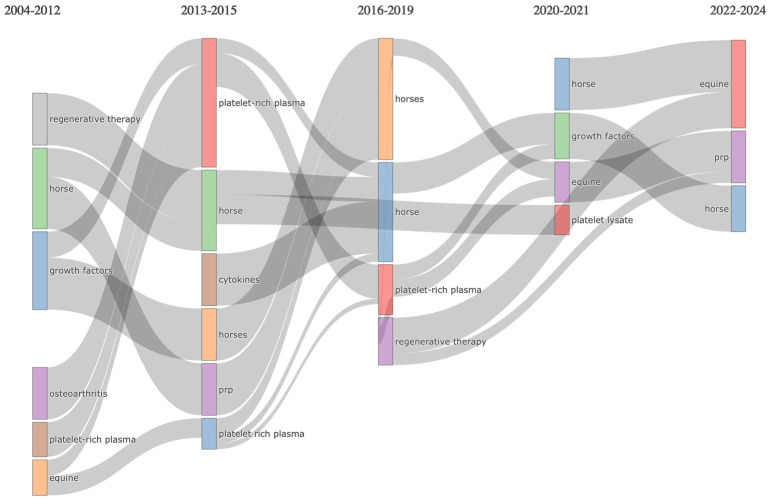
Connection plot of the thematic evolution of author keywords (2000–2024).

The general thematic evolution of the author keywords between 2000 and 2024 is shown in Figure 10. The evolution of keywords considering the generation of clusters according to niche themes, motor themes, basic themes, and emerging or declining themes by cutting year (2012, 2015, 2019, and 2021) is shown in Table 3.

### 3.9. Social Structure

**Figure 11:**
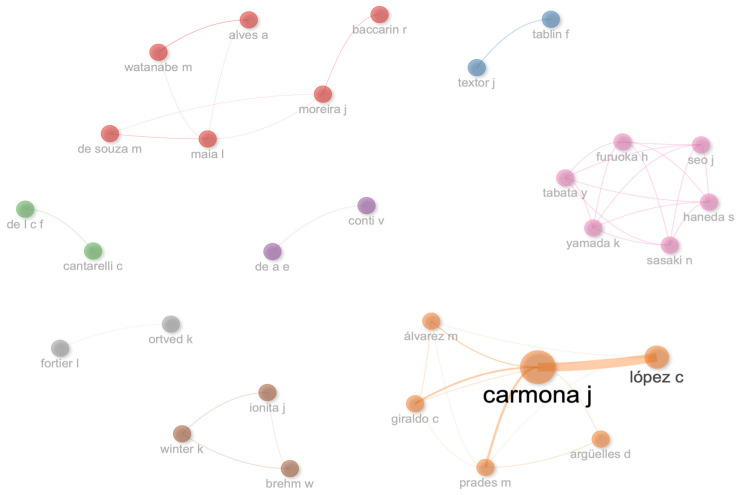
Collaboration network for authors.

**Figure 12:**
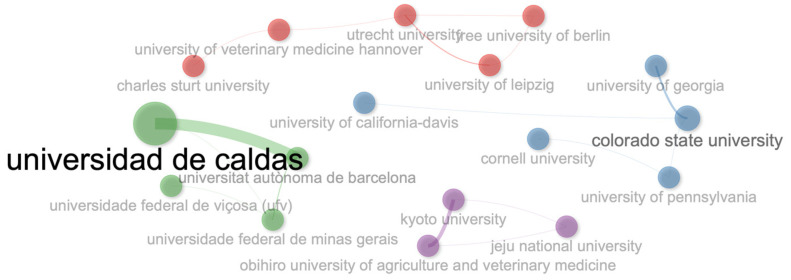
Collaboration network for institutions.

**Figure 13:**
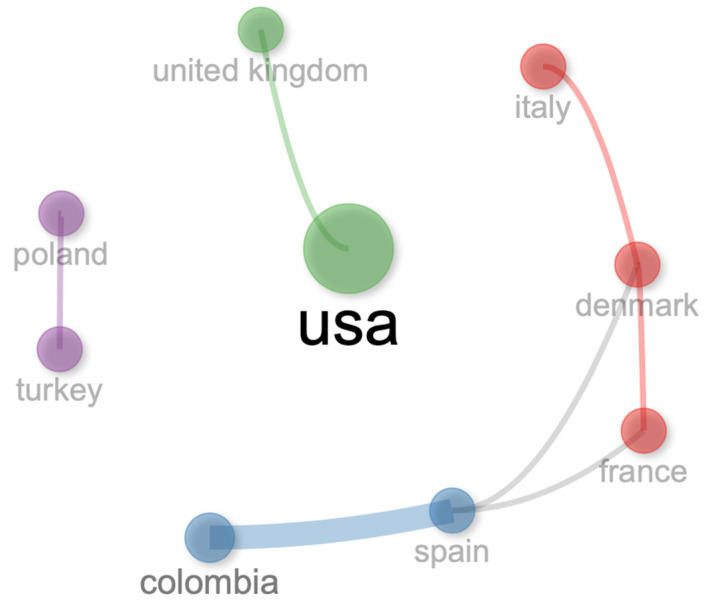
Collaboration network for countries.

The collaboration network of this bibliometric analysis for authors (Figure 11), institutions (Figure 12), and countries (Figure 13) is isolated and small. In general, it can be noted that there is a scarce collaboration between researchers, institutions, and countries, and the development of this body of research could be isolated with different perceptions about this topic in the function of researchers.

## 4. Discussion

Bibliometric analysis, also known as systematic scientometric review, is a computational methodology useful for identifying the most relevant set of peer-reviewed publications on a given topic. In addition, this methodology can be used to understand various aspects of research, such as trends, hotspots, and gaps in knowledge within a given scientific field [51]. This computational tool makes it possible to assess the scientific contribution of authors and their institutions in different countries and how they are connected to researchers in other countries and even in different research fields [52,53].

To the authors’ knowledge, this is the first bibliometric study to evaluate the scientific production in the field of the use of PRP and related orthobiologics, such as ACS, APS, and platelet lysates, among others, for the treatment of chronic musculoskeletal disorders in horses. However, there are several studies evaluating the bibliometric impact on the use of PRP in human orthopedics [53,54,55,56,57].

On the other hand, the present study may be the first to use information from two major databases (WOS and Scopus), using the *merge()* function of the R package to remove duplicate records from these databases. This action allowed the integration of the two databases and the obtaining of a greater number of records to improve the power of this bibliometric analysis. Furthermore, it is important to note that a manual review of the records is essential to screen the registries and to avoid the inclusion of documents that could alter the final results of the bibliometric study.

This analysis showed that the scientific production of this field of research is mainly published in four journals (sources). This fact could be confirmed by applying Bradford’s law [58], which states that the majority of documents on a given topic could be published in a reduced number of journals dedicated to that particular research topic, together with certain marginal sources and many other general or dispersed journals. Therefore, the journals focused on a given research area could act as a family of successive generations of decreasing relatedness, with each generation larger than the previous one. The practical application of Bradford’s law provides the mechanisms to select the journals that are not only the most productive but also the most relevant to cover a given area of knowledge [59].

In terms of authors’ production, according to the number of articles published, the number of citations, and the H-index impact, researchers from Colombia and Spain lead the top 10 lists. This fact could be confirmed by Lotka’s law, a bibliometric law that postulates that most of the authors publish a small number of documents, while a few authors publish the majority of relevant documents in a given research field [60].

The most productive institutions in this research area were Universidad de Caldas, Colorado State University, University of California-Davis, and University of Leipzig. This production coincides to some extent with the production of authors. However, when academic production is measured in terms of countries, the United States, Brazil, and Colombia yield the most production. This fact can be explained by the fact that the USA and Brazil have a large number of researchers and institutions that produce scientific literature in this field, compared to Colombia, where only a single institution (Universidad de Caldas) is responsible for producing research on this topic.

The most frequently used author keywords were horse, platelet-rich plasma, equine, osteoarthritis, and autologous conditioned serum. The size of the keyword “platelet-rich plasma” in the cloud plot could be larger because other keywords such as “platelet rich plasma” and “prp” have the same meaning as “platelet-rich plasma”. It is important to consider that the author keywords are closely related to the trending topics in this field of research, where new investigations on platelet lysates appear or the term “orthobiologic” begins to be used.

Clustering by coupling analysis revealed three major clusters that integrate the research in this area. In general, one cluster focuses on basic studies measuring the concentration of growth factors in equine platelet-rich gel supernatants [61,62]. Another cluster of investigations focused on the role of PRP and ACS as treatments for OA [14,32], while another cluster emphasized the effect of PRP on bone marrow and mesenchymal stem cells [45,63]. On the other hand, the conceptual structure analysis evidenced three primary author keywords (horse, platelet-rich plasma, and osteoarthritis) clusters around which secondary keywords revolved, such as biologics, arthroscopy, tendon, sport medicine, and so on.

Regarding the thematic evolution of this bibliometric analysis, few changes could be observed in the use of author keywords over time, although a large number of author keywords were used during 2023–2015 and 2016–2019, indicating that scientific production was more extensive and diverse during these periods. This fact could be corroborated by analyzing the evolution of the cluster map by cutting year, which could indicate that the research interest in this field is either decreasing in or has been concentrating on specific topics (clusters) over the last five years.

The analysis of the collaboration network in terms of authors, institutions, and countries of this bibliometric analysis indicates an isolated development of individual author networks, with modest collaborations between institutions and countries. This fact could indicate the necessity of developing joint research on the use of platelet-rich plasma and related hemocomponents for the treatment of chronic musculoskeletal disorders in horses to consolidate the knowledge obtained and to open new avenues of investigations in this field. Furthermore, this situation demonstrates that the great disparity in the use of author keywords and the formation of non-related clusters could be a symptom of the scarce association of the main factors (authors, institutions, and countries) that shape this field of veterinary science.

## 5. Conclusions

This bibliometric study carried out between 2000 and 2024 allows us to conclude that the academic production of documents (articles and reviews) in the field of the use of PRP and related orthobiologics for the treatment of musculoskeletal disorders in horses has been mainly published in four journals (*Frontiers in Veterinary Science*, *Journal of Equine Veterinary Science*, *BMC Veterinary Research*, and the *American Journal of Veterinary Research*). The most productive institutions on this topic were Universidad de Caldas, Colorado State University, University of California-Davis, and University of Leipzig, and the most productive countries were the USA, Brazil, and Colombia. Horse, platelet-rich plasma, equine, osteoarthritis, and autologous conditioned serum were the most common keywords used by the authors. The trending topics in this area are platelet lysates and orthobiologics. It is possible that the research interest in this field has either decreased or concentrated on specific topics in the last 5 years. The collaboration network of authors, institutions, and countries shows an isolated development of individual author networks with modest collaboration between institutions and countries.

## Figures and Tables

**Table 1:** Top ten core sources according to Bradford’s law.

Source	Rank	Frequency	Cumulative Frequency	Zone
Frontiers in Veterinary Science	1	26	26	Zone 1
Journal of Equine Veterinary Science	2	24	50	Zone 1
BMC Veterinary Research	3	11	61	Zone 1
Equine Veterinary Journal	4	11	72	Zone 1
Pferdeheilkunde	5	11	83	Zone 2
American Journal of Veterinary Research	6	10	93	Zone 2
Archivos de Medicina Veterinaria	7	10	103	Zone 2
Veterinary Clinics of North America—Equine Practice	8	8	111	Zone 2
Equine Veterinary Education	9	7	118	Zone 2
Research in Veterinary Science	10	7	125	Zone 2

**Table 2:** Top 10 most global cited documents on PRP and related orthobiologicals as treatments for equine musculoskeletal disorders (2000–2024).

Authors	Source	Topic	DT	TC	TCPY	NTC
Frisbie et al. [43]	American Journal of Veterinary Research	Autologous conditioned serum (ACS) and experimental osteoarthritis (OA)	Article	207	11.50	1.90
Waselau et al. [44]	Journal of the American Veterinary Medical Association	Platelet-rich plasma (PRP) and suspensory ligament desmitis	Article	111	6.53	2.14
Del Bue et al. [45]	Veterinary Research Communications	PRP plus mesenchymal stem cells and tendonitis	Article	94	5.53	1.81
Argüelles et al. [46]	Research in Veterinary Science	PRP technique	Article	93	4.89	1.00
Fortier et al. [46]	Veterinary Clinics of North America—Equine Practice	Regenerative medicine and tenodesmic lesions	Review	93	5.47	1.79
Carmona et al. [47]	Journal of Equine Veterinary Science	PRP and OA	Article	80	4.44	0.74
Sutter et al. [42]	American Journal of Veterinary Research	PRP technique	Article	78	3.71	1.00
Hraha et al. [48]	Equine Veterinary Journal	ACS and IRAP techniques	Article	78	5.43	3.21
Textor et al. [49]	Veterinary Surgery	PRP and exogenous activation	Article	76	5.85	3.06
Argüelles et al. [50]	Veterinary Record	PRP and tenodesmic lesions	Article	75	4.41	1.45

DT = document type; TC = total citations; TCPY = total citations per year; NTC = normalized total citations.

**Table 3:** General thematic evolution of author keywords by cutting year (2012, 2015, 2019, and 2021) according to four themes.

Cutting Year	Niche Themes	Motor Themes	Basic Themes	Emerging or Declining Themes
2012	Cluster 1 (growth factors, horse, collagenase)	Cluster 1 (horse, tendon, prp) Cluster 2 (equine, platelet-rich plasma, mesenchymal stem cells)	Cluster 1 (platelet-rich plasma, bone marrow, autologous conditioned serum)	Cluster 1 (regenerative therapy) Cluster 2 (platelet)
2015	Cluster 1 (comparison, prp). Cluster 2 (platelet rich plasma, mesenchymal stem cell, tissue engineering)	Cluster 1 (horse, platelet concentrate, regenerative therapy). Cluster 2 (platelet rich plasma, mesenchymal stem cell, tissue engineering). Cluster 3 (osteoarthritis, autologous conditioned serum, stem cells)	Cluster 1 (platelet-rich plasma, equine, growth factors). Cluster 2 (osteoarthritis, autologous conditioned serum, stem cells)	Cluster 1 (cytokines). Cluster 2 (horses)
2019	Cluster 1 (cryopreservation, dmso, platelet-rich plasma (prp)). Cluster 2 (osteoarthritis, cartilage, aggrecan) Cluster 3 (ultrasound) Cluster 4 (autologous conditioned serum)	Cluster 1 (platelet-rich plasma, regenerative medicine, tendon) Cluster 2 (osteoarthritis, cartilage, aggrecan) Cluster 3 (growth factors, regenerative therapy, cytokines)	Cluster 1 (equine, mesenchymal stem cells, stem cells). Cluster 2 (growth factors, regenerative therapy, cytokines) Cluster 3 (horse, autologous conditioned serum, acs) Cluster 4 (horses, platelet rich plasma (prp)	Cluster 1 (growth factor). Cluster 2 (growth factors, regenerative therapy, cytokines)
2021	Cluster 1 (growth factors, prp, healing)	Cluster 1 (equine, sports medicine, biological therapies) Cluster 2 (horse, osteoarthritis, autologous conditioned serum)	Cluster 1 (platelet-rich plasma) Cluster 2 (horse, osteoarthritis, autologous conditioned serum)	Cluster 1 (platelet-rich plasma) Cluster 2 (platelet lysate)

## Data Availability

The original contributions presented in this study are included in the article. Further inquiries can be directed to the corresponding author.
